# Drainage volume on postoperative day one to predict clinically relevant postoperative pancreatic fistula following distal pancreatectomy

**DOI:** 10.1186/s12893-022-01748-z

**Published:** 2022-08-01

**Authors:** Quanyu Zhou, Wei He, Yao liu, Bo liao, Yong Liang, Bing Mo, Shujun Yin, Weian Tang, Yuhong Shi, Yuxiao Xia

**Affiliations:** 1 Department of Hepatobiliary and Pancreatic Surgery, The Affilliated Chengdu 363 Hospital of Southwest Medical University, Chengdu, Sichuan 610041 People’s Republic of China; 2 Department of Nuclear Medicine, Second Affiliated Hospital of Chengdu Medical College (China National Nuclear Corporation 416 Hospital), Chengdu, Sichuan 610000 People’s Republic of China

**Keywords:** Distal pancreatectomy, Postoperative pancreatic fistula, The drainage volume on the first postoperative day

## Abstract

**Background:**

The purpose of this study was to determine how the drain fluid volume on the first day after surgery (DFV 1) can be used to predict clinically relevant post-operative pancreatic fistula following distal pancreatectomy (DP).

**Method:**

A retrospective analysis of 175 patients who underwent distal pancreatectomy in hepatobiliary surgery at Chengdu 363 Hospital (China) from January 2015 to January 2021 has been performed. Depending on the presence of pancreatic fistula, all patients were divided into two groups: POPF and non-POPF. The clinical factors were analyzed using SPSS 17.0 and Medcalc software. In order to assess the effectiveness of DFV 1 in predicting POPF after surgery, ROC curves were used to calculate its cut-off point,, which yielded sensitivity and negative predictive value of 100% for excluding POPF.

**Result:**

Of the 175 patients who underwent distal pancreatectomy, the incidence of overall pancreatic fistula was 36%, but the rate of clinically significant (grade B and C) fistula, as defined by the International Study Group on Pancreatic Fistula, 30 was only 17.1% (28 grade B and 2 grade C fistula). The results from univariate and multivariate logistic regression analysis showed that drain fluid volume on the first postoperative day (OR = 0.95, *P* = 0.03), drainage fluid amylase level on POD1 (OR = 0.99, *P* = 0.01) and the preoperative ALT level (OR = 0.73, *P* = 0.02) were independent risk factors associated with CR-POPF. Receiver operating characteristic (ROC) curve analysis revealed that a drainage volume of 156 mL within 24 h and an amylase greater than 3219.2 U/L on the first postoperative day were the optimal thresholds associated with complications.

**Conclusion:**

After distal pancreatectomy, the drainage volume on the first postoperative day can predict the presence of a clinically relevant pancreatic fistula.

## Introduction

Distal pancreatectomy (DP) was first performed by Billroth in 1884 and was further outlined by Mayo in 1913 [1]. This operative is the standard procedure for tumors located in the body or tail of the pancreas, such as pancreatic carcinoma, intraductal papillary mucinous neoplasms, neuroendocrine tumors, and invasive carcinomas from other organ carcinomas including stomach and colon [2]. Postoperative pancreatic fistula (POPF) is one of the most common complications of pancreatic surgery [3]. This complication, as defined by the International Study Group for Pancreatic Fistulas (ISGPF), is divided into two main groups: Clinically irrelevant fistulas (i.g., biochemical leaks) as well as clinically relevant pancreatic fistulas (CR-POPFs) requiring postoperative management adaptations (i.e., grades B and C) [4]. The resulting leakage of pancreatic effluent can lead to significant morbidity characterized by deep organ space infection, hemorrhage, end organ failure, and even death [5]. Thanks to advances in surgical techniques and perioperative management, the mortality rate of DP has declined to below 3% in high-volume centers. However, there are still reports of postoperative pancreatic fistula (POPF) occurring as high as 10% [6]. We retrospectively collected the clinical data of patients undergoing distal pancreatectomy at the Chengdu 363 Hospital (China) in the past five years. Investigate the prognostic factors of clinically related pancreatic fistulas after distal pancreatectomy, and to determine how the drain fluid volume on the first day after surgery (DFV 1) can be used to predict clinically relevant post-operative pancreatic fistula following distal pancreatectomy (DP).

## Materials and methods

### General information

A retrospective analysis of 175 patients who underwent distal pancreatectomy in hepatobiliary surgery at Chengdu 363 Hospital (China) from January 2015 to January 2021 has been performed. The patients’ characteristics, including age, sex, body mass index (BMI), abdominal symptom, history of abdominal operations, American Society of Anaesthesiologists (ASA) grading, surgical method, pancreatic stump treatment method, time spent operating, amount of blood lost, amount of transfusions during and after surgery, dimensions of the main pancreatic duct, width of the pancreatic stump, serological examination before and on the first day after surgery, drain fluid volume on the first day after surgery (DFV 1), duration of hospital stay, case type and tumor size, etc. were recorded. The diameter of the main pancreatic duct and the width of the pancreatic stump were obtained by two researchers respectively measured on preoperative imaging (mostly CT) data and then taking the average value. See Table 1.

**Table 1:** Univariate regression analysis of POPF risk factors for patients (Continues variables)

Characteristics	POPF groups (n=30)	non-POPF groups (n=145)	*t*	*P* value
Age (Year)	58.56±12.18	56.42±15.79	0.69	0.48
BMI (kg/m^2^)	24.62±4.87	22.37±3.35	2.40	0.02
Hb (g/L)	121.70±16.02	125.78±17.69	-1.16	0.244
ALT (IU/L)	29.53±16.12	23.16±15.15	2.07	0.04
AST (IU/L)	23.20±8.20	23.12±10.27	0.04	0.96
Prealbumin (mg/L)	264.96±51.92	240.24±4.80	5.67	0.001
Total Protein (g/L)	69.90±6.89	67.70±11.34	1.39	0.16
Albumin (g/L)	41.96±5.02	42.19±4.66	− 0.23	0.81
Globulin (g/L)	27.43±4.53	27.95±6.67	− 0.56	0.57
Operation time (min)	233.20±83.0	236.37±70.31	-0.21	0.82
Bleeding (ml)	456.30±266.97	512.02±393.67	-0.94	0.34
Prealbumin (mg/L the first day after the surgery)	196.23±50.65	173.90±47.52	2.31	0.02
Albumin (g/L the first day after the surgery)	34.50±6.44	36.16±7.11	− 1.18	0.23
Globulin (g/L the first day after the surgery)	22.63±3.22	23.23±3.75	− 0.81	0.41
Blood glucose (mmol/L the first day after the surgery)	11.20±2.73	9.51±3.80	2.30	0.02
Drainage on the first day after surgery (ml)	158.20±105.36	113.42±63.72	2.24	0.03
Drainage fluid amylase level on POD1 (U/L)	6979±3742	2521±2285	8.59	0.001
The main pancreatic duct (cm)	0.21±0.10	0.19±0.09	0.66	0.50
Anteroposterior diameter of pancreatic stump (cm)	2.09±0.41	1.92±0.49	1.73	0.08
Tumor size (cm)	5.77±2.40	4.84±2.59	1.80	0.72

### Surgical techniques

Patients complicated with combined organ resection, severe systemic diseases with life expectancy of < 1 year, and emergency surgery were not included in the research. All patients in our study underwent elective pancreatic body and tail resection (with or without spleen preservation). Of these 175 patients, 36 patients underwent laparoscopic pancreatectomy and 139 patients underwent open laparotomies. Distal pancreatectomy (DP) was performed with the standard procedure. We approached pancreatic stump in three ways, including 120 cases of linear incision closure, 30 cases of electrosurgical dissection, and 25 cases of electrosurgical dissection and main pancreatic duct ligation. The stumps of the above three methods were all used for interrupted polypropylene suture reinforcement (Prolene; Ethicon Products, USA).

Preoperative antibiotic prophylaxis consisted of a preoperative dose, an intraoperative dose, and three postoperative doses of Ceftizoxime (3 g intravenous).

### Pancreatic Fistula Definition

In accordance with clinical impact on a patient's hospital course, the ISGPS has defined three levels of POPF (biochemical leak, grades B and C) [4]. In biochemical leak, the amylase level in the drainage fluid is more than three times what is normally present in the serum postoperatively. In Grade B, patients usually experience abdominal pain, fever, and/or leukocytosis; antibiotics are usually prescribed, and somatostatin analogues are also sometimes used. Patients are often fasting and receiving parenteral or enteral nutrition which results in a delayed discharge because of the necessity of parenteral or enteral nutrition. The condition of grade C POPF is formed when organ failure or clinical instability occurs in a patient with grade B POPF. This condition often requires a reoperative procedure, prolonged hospital stay, intensive care unit stay, and sometimes even results in death [7, 8]. In our study, grade B and grade C were defined as clinically relevant pancreatic fistula (CR-POPF).

### Statistical analysis

Data were analyzed using SPSS software (version 17.0, SPSS, Inc., Chicago, IL, USA) and MedCalc for Windows (version 14.8.1, MedCalc Software, Ostend, Belgium) [9]. Univariate analysis of continuous variables was performed using Students' *t* tests, and categorical variables were analyzed using Chi-square tests. Factors for which *P* < 0.20 in univariate analysis were selected for multiple logistic regression analysis. By analyzing the ROC curve, the effectiveness of DFV1 at predicting POPF after surgery was determined and the cut-off value was determined. An area under the curve (AUC) calculation was performed, and an AUC > 0.5 was deemed significant in diagnosing. All *P* values were two sided, and* P* < 0.05 was considered statistically significant.

## Results

### Patient demographics and postoperative complications

The current study enrolled 175 patients from January 2015 to January 2021. The population consisted of 79 males and 96 females, ranging in age from 23 to 78 years, with a mean age of 56 ± 15 years. Body mass index between 12.9 and 37.3 kg/m^2^ with an average BMI of 22.75 ± 3.74 kg/m^2^. The participants received an average of 235.83 ± 5.47 min (range: 83 to 560 min). The mean amount of intraoperative bleeding was 516.82 ± 26.82 ml (range: 50 to 1723 ml). The preoperative ALT level ranged from 3 to 76 IU/L, with an average of (24.26 ± 15.46) IU/L. The prealbumin level was 145–382 mg/L, with an average of (244.49 ± 23.57) mg/L. A total of 41 of the patients had a history of hypertension, 35 had diabetes mellitus, and 34 with a past history of upper abdominal surgery. Based on ASA score, there were 123 cases with score (≤ 2) and 52 cases with score (> 2). The diameter of the main pancreatic duct was 0.1 to 1.1 cm, with an average of (1.95 ± 0.48) cm. In Tables 1 and 2, the full demographic and clinical data of patients were displayed.

**Table 2:** Univariate regression analysis of POPF risk factors for patients (categorical variables)

Characteristics	n (175)	POPF (30)	${X}^{2}$	*P* value
Gender				
Male	79 (45%)	15 (50%)	0.34	0.55
Female	96 (45%)	15 (50%)		
Abdominal symptom				
Presence	107 (61%)	19 (63%)	0.07	0.78
Absence	68 (39%)	11 (37%)		
Hypertension				
Presence	41 (23%)	8 (26.6%)	0.21	0.64
Absence	134 (77%)	22 (73.3%)		
Diabetes				
Presence	35 (20%)	10 (33.3%)	2.00	0.04
Absence	140 (80%)	20 (66.6%)		
Previous abdominal surgery				
Presence	34 (19%)	5 (16.6%)	0.17	0.67
Absence	141 (81%)	25 (83.3%)		
ASA score				
Score (≤2)	123 (70%)	15 (50%)	7.13	0.01
Score (>2)	52 (30%)	15 (50%)		
Surgical techniques				
Laparoscopic spleen-preserving	17 (9.7%)	1 (3.3%)	3.62	0.30
Laparoscopic splenectomy	19 (10.8%)	2 (6.6%)		
Open spleen-preserving	18 (10.2%)	5 (16.6%)		
Open splenectomy	121 (69.1%)	22 (73.3%)		
Management of the pancreatic stump				
Linear incision closure	120 (68.5%)	26 (86.6%)	4.42	0.10
Electrosurgical dissection	30 (17.1%)	3 (10%)		
Electrosurgical dissection and main pancreatic duct ligation	24 (13.7%)	1 (3.3%)		
Cystadenoma	64 (36.5%)	9 (30%)		
Pseudopapillary neoplasm	14 (8%)	4 (13.3%)		
Pathological type				
Pancreatic cancer	63 (36%)	11 (36.6%)	1.07	0.78
Neuroendocrine tumors	24 (13.7%)	4 (13.3%)		
Other	9 (5%)	2 (6.6%)		

### Occurrence of clinically relevant postoperative pancreatic fistula (POPF)

Of the 175 patients who underwent distal pancreatectomy, the incidence of overall pancreatic fistula was 36%, but the rate of clinically significant (grade B and C) fistula, as defined by the International Study Group on Pancreatic Fistula, 30 was only 17.1% (28 grade B and 2 grade C fistula). For patients with Grade B pancreatic fistula, percutaneous drainage of an intra-abdominal collection was carried out in 10 patients because of encapsulated peritoneal effusion. Four cases developed abdominal infection and septicemia, and the condition improved after anti-infective treatment. Six patients with postoperative drainage tube indwelling for more than three weeks. Secondary surgical procedures were performed in 2 cases of grade C pancreatic fistula patients. Compared to most previous studies, the rate of POPF in this study is lower [10]. Fortunately, no postoperative mortality occurred in this study.

### Risk factors for pancreatic fistula following DP in univariate analysis

There were significant relationships between POPF and these factors according to the univariate analysis: BMI (*P* = 0.02), Preoperative ALT level (*P* = 0.04), Prealbumin level before surgery and 1 day after surgery (with *P* values of 0.001, 0.02, respectively), Drainage on the first day after surgery (*P* = 0.03), Drainage fluid amylase level on POD1 (*P* = 0.001), Glucose levels on the morning of the first postoperative day (*P* = 0.02), History of diabetes mellitus (*P* = 0.04), ASA score > 2 (*P* = 0.01).

### Using multivariate analysis to analyze pancreatic fistula risks after DP

A multivariate logistic regression analysis was performed on the risk factors found in univariate logistic regression analyses. Based on results of multivariable logistic regression, that drain fluid volume on the first postoperative day (OR = 0.95, *P* = 0.03), drainage fluid amylase level on POD1 (OR = 0.99, *P* = 0.01) and the preoperative ALT level (OR = 0.73, *P* = 0.02) were independent risk factors for clinically relevant (See Table 3).

**Table 3:** Multivariate regression analysis of POPF risk factors for patients

Variables	Odds ratio	95% CI	*P* value
BMI (kg/m^2^)	0.98	0.94–1.02	0.52
Diabetes	0.34	0.09–1.32	0.12
ALT (IU/L)	0.73	0.60–0.89	0.02
Prealbumin (mg/L)	0.99	0.98–1.00	0.14
ASA score	0.50	0.12–1.95	0.32
Prealbumin (mg/L the first day after the surgery)	0.99	0.98–1.01	0.65
Blood glucose (mmol/L the first day after the surgery)	0.93	0.77–1.12	0.46
Drainage on the first day after surgery (ml)	0.95	0.92–0.98	0.03
Drainage fluid amylase level on POD1 (U/L)	0.99	0.99–1.00	0.01

### ROC Analyses

**Fig. 1:**
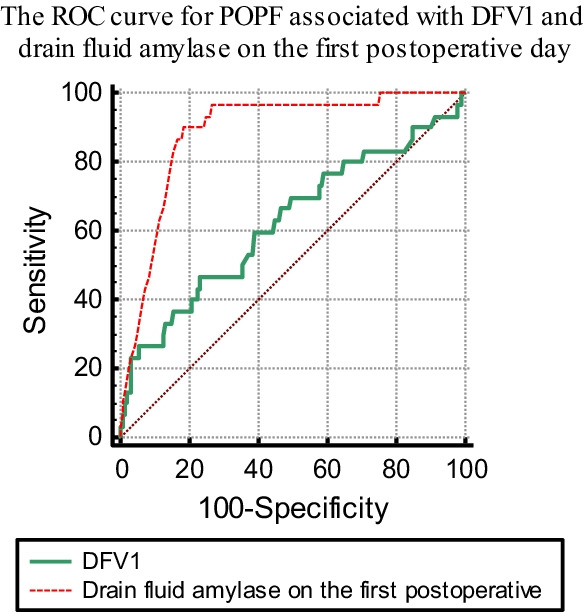
The ROC curve for POPF associated with DFV1 and drain fluid amylase on the first postoperative day

In determining CR-POPF discrimination thresholds. Based on the receiver operating characteristic (ROC) curve, the optimal threshold for the association between drainage volume and complications was 156 mL in 24 h. It has an area under the curve of 0.624 (95% CI: 0.548–0.696). It measures sensitivity and specificity at 46.67% and 76.55%, respectively. Receiver operating characteristic curve analysis of drain fluid amylase on the first postoperative day. A cut-off value of 3219.2 U/L was associated with 90% sensitivity, 81.38% specificity for POPF (AUC: 0.885, 95% CI: 0.829–0.928). see Fig. 1.

## Discussion

Distal pancreatectomy (DP) is the standard procedure for tumors located in the body or tail of the pancreas [2]. The digestive pancreatic enzymes leak out of the pancreatic ductal system via an abnormal connection into the peri-pancreatic space or the peritoneal cavity, with resulting morbidity such as abdominal pain, ileus, fever, and the possibility of abscess, sepsis, and hemorrhage and consequently prolonged hospitalization [11]. Thanks to advances in surgical techniques and perioperative management, the mortality rate of DP has declined to below 3% in high-volume centers. However, there are still reports of postoperative pancreatic fistula (POPF) occurring as high as 10% [6]. Even though extensive efforts have been made to reduce the occurrence of postoperative pancreatic fistulas, there has not been any noticeable improvement since the early 1970s [12, 13]. Therefore, to reduce the high mortality rate associated with pancreatic fistula and its complications, it is of great importance to understand the risk factors for pancreatic fistula. And to determine how the drain fluid volume on the first day after surgery (DFV 1) can be used to predict clinically relevant post-operative pancreatic fistula following distal pancreatectomy (DP).

The present study shows that preoperative ALT level was independent risk factors for clinically relevant pancreatic leakage (*P* < 0.05). Hepatocellular injury often results in an increase in serum ALT, and serum ALT levels are used as a marker for liver injury. We speculate that patient with impaired liver function as more prone to develop POPF and consequently have more drain fluid volume.

Univariate analysis in this study showed that patients with higher prealbumin levels had a higher incidence of postoperative POPF. Previous research has indicated that low albumin levels in the preoperative serum are an independent risk factor for major postoperative complications. It is also one of the leading causes of postoperative mortality [14, 15]. An abnormally low serum albumin level is an indication that the patient is nutritionally deficient or that his liver is damaged. This leads to a constant plasma colloid osmotic pressure, while hypoproteinemia impairs water balance, increasing the likelihood of hypovolemia. This suggests that such patients are at higher risk of sustaining a complication from surgery. We suppose that prealbumin is the precursor to albumin. Because a negative feedback regulatory mechanism increases the level of prealbumin. Therefore, preoperative hypoproteinemia needs to be taken into consideration and necessary treatment administered. This means not only raising the preoperative serum albumin level, but also improving comprehensively the nutritional state of the patients before surgery to improve their recovery.

A meta-analysis and several large-cohort studies have proven that drain fluid amylase on the first postoperative day is a good predictor of development of pancreatic fistula [16–18]. The results of this study showed that the AUC of the amylase level in the drainage fluid on the first postoperative day for predicting POPF was as high as 0.885, with a sensitivity and specificity of 90% and 81.38%, respectively. These results are in agreement with previous literature. In this paper, the indicators on the first day after surgery were systematically analyzed, and it was concluded that the blood glucose level and DFV1 on the first day after surgery were high-risk factors for pancreatic leakage. And there was an independent association between DFV1 and clinically relevant pancreatic leakage after DP. We believed that it may be because the higher DFV1 indicates that the pancreatic stump exudates more, which will affect the healing of the pancreatic stump, thus leading to the occurrence of POPF. In agreement with this notion, Uchida et al. demonstrated that Postoperative complications, especially clinically relevant postoperative pancreatic fistula, are significantly associated with large and heterogeneous collections of peripancreatic tissue. It would be possible to safely collect a small and homogenous part of the peripancreatic tissue [19]. We further performed ROC curve analysis to develop a cut-off value for postoperative drainage volume to predict postoperative pancreatic fistula. The results showed that when DFV 1 > 156 ml, the incidence of CR-POPF increased significantly (*P* = 0.036). An average sensitivity and specificity of 46.67% and 76.55% were achieved respectively. see Fig. 1.

A prospective randomized multicenter trial conducted by Van Buren et al. showed that clinical outcomes are similar in DP with and without intraperitoneal drainage. In their opinion, routine prophylactic drainage after pancreatic resection was not necessary [20]. However, in a meta-analysis by Lu Huan et al., it was found that patients who underwent DP could choose to skip the drainage. In their opinion, abdominal drainage would induce the wound to heal slowly. Because of the closed suction system, abdominal infection and post-operative pancreatic fistulas (POPF) may occur [21]. However, the above views have not yet reached a consensus. According to the results of this paper, the DFV1 is an independent risk factor associated with POPF. The drainage can find the POPF, hemorrhage, biliary fistula, peritoneal fluid collection, and so on after DP. Therefore, we suggest that drainage tubes should be placed routinely after surgery, and early removal should be determined according to DFV 1 and the combination of the above-mentioned high-risk factors. When DFV 1 > 156 ml, the occurrence of postoperative pancreatic leakage should be more vigilant (*P* = 0.036). Somatostatin can be continuously pumped in advance or the abdominal CT can be closely reviewed to detect and deal with the problem as soon as possible.

## Conclusions

It is safe to conclude that the development of a POPF is the most common and potentially life-threatening surgical complication following a distal pancreatectomy.

The present study shows that drain fluid volume on the first postoperative day, drainage fluid amylase level on POD1 and the preoperative ALT level were independent risk factors for clinically relevant pancreatic leakage (*P* < 0.05). The results of this analysis, however, remain subject to large samples, multicenter collaboration, and further randomized controlled trials.

## Data Availability

The datasets used and/or analyzed during the current study are available from the corresponding author on reasonable request (Email: hahaxiayuxiao@163.com).

## Abbreviations

DP: Distal pancreatectomy
POPF: Postoperative pancreatic fistula
ISGPF: International Study Group for Pancreatic Fistula
CR-POPF: Clinically relevant pancreatic fistula
ASA: American Society of Anaesthesiologists grading
DFV 1: Drain fluid volume on the first day after surgery
ISGPS: International Study Group on Pancreatic Surgery
